# Prediction of Thermochemical Properties of Long-Chain Alkanes Using Linear Regression: Application to Hydroisomerization

**DOI:** 10.1021/acs.jpcb.4c05355

**Published:** 2024-09-23

**Authors:** Shrinjay Sharma, Josh J. Sleijfer, Jeroen Op de Beek, Stach van der Zeeuw, Daniil Zorzos, Silvia Lasala, Marcello S. Rigutto, Erik Zuidema, Umang Agarwal, Richard Baur, Sofia Calero, David Dubbeldam, Thijs J.H. Vlugt

**Affiliations:** † Engineering Thermodynamics, Process & Energy Department, Faculty of Mechanical Engineering, Delft University of Technology, Leeghwaterstraat 39, 2628CB Delft, The Netherlands; ‡ Delft Institute of Applied Mathematics, Faculty of Electrical Engineering, Mathematics and Computer Science, Delft University of Technology, Mekelweg 4, 2628CD Delft, The Netherlands; § Faculty of Applied Sciences, Delft University of Technology, Lorentzweg 1, 2628 CJ Delft, The Netherlands; ∥ Faculty of Aerospace Engineering, Delft University of Technology, Kluyverweg 1, 2629 HS Delft, The Netherlands; ⊥ Université de Lorraine, CNRS, LRGP, F-54000 Nancy, France; # Shell Global Solutions International B.V., Grasweg 39, 1031HW Amsterdam, The Netherlands; ∇ Shell Chemical LP, Monaca, Pennsylvania 15061, United States; ○ Department of Applied Physics, Eindhoven University of Technology, 5600MB Eindhoven, The Netherlands; ◆ Van’t Hoff Institute of Molecular Sciences, University of Amsterdam, Science Park 904, 1098XH Amsterdam, The Netherlands

## Abstract

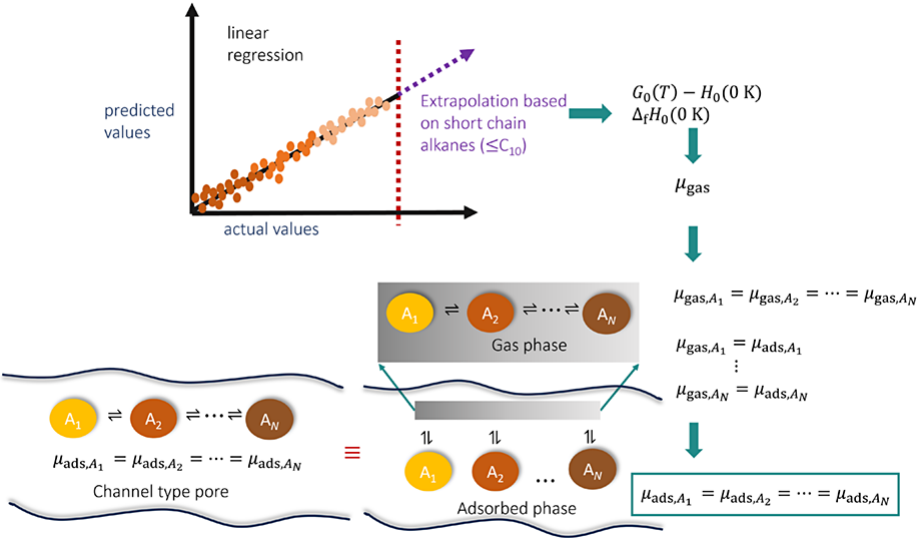

Linear regression (LR) is used to predict thermochemical properties of alkanes at temperatures (0–1000) K to study chemical reaction equilibria inside zeolites. The thermochemical properties of C_1_ until C_10_ isomers reported by Scott are used as training data sets in the LR model which is used to predict these properties for alkanes longer than C_10_ isomers. Second-order groups are used as independent variables which account for the interactions between the neighboring groups of atoms. This model accurately predicts Gibbs free energies, enthalpies, Gibbs free energies of formation, and enthalpies of formation for alkanes which exceeds the chemical accuracy of 1 kcal/mol and outperforms the group contribution methods developed by Benson et al., Joback and Reid, and Constantinou and Gani. Predictions from our model are used to compute the reaction equilibrium distribution of hydroisomerization of C_10_ and C_14_ isomers in MTW-type zeolite. Calculation of reaction equilibrium distribution inside zeolites also requires Henry coefficients of the isomers which can be computed using classical force field-based molecular simulations using the RASPA2 software for which we created an automated workflow. The reaction equilibrium distribution for C_10_ isomers obtained using the LR model and the training data set for this model are in very good agreement. The tools developed in this study will enable the computational study of hydroisomerization of long-chain alkanes (>C_10_).

## 1 Introduction

In transitioning toward fuels and chemicals from renewable sources, platforms that provide clean hydrocarbon liquid energy carriers from carbon dioxide directly or via biocomponents can play an important role.^1^ For sustainable aviation fuel and low carbon gas-oil or lubricants, iso-alkanes with a high degree of branching are the preferred constituents,^2^ and hence shape-selective zeolite catalyzed hydroisomerization,^3^ often called catalytic dewaxing, is likely to become a key step in the production of iso-alkanes, as it currently is for the classical analogue products.^4,5^ To design processes, catalysts and equipment handling, reacting and separating (iso)alkanes and their mixtures, a detailed understanding of the thermochemical properties of these compounds, such as enthalpy, Gibbs free energy, entropy, heat capacity, and fugacity, is necessary.^6^ To compute a reliable product distribution from hydroisomerization reactions, accurate prediction of thermochemical properties of alkanes is of utmost importance.^7^ Another example of interest is alkane-based phase change materials for thermal energy storage systems.^8^

Thermochemical properties of all isomers until C_10_ are reported by Scott.^9^ The work of Scott lists Gibbs free energies (*G*_0_ – *H*_0_(0 K)), enthalpies (*H*_0_ – *H*_0_(0 K)), absolute entropies *S*_0_, Gibbs free energies of formation Δ_f_*G*_0_, enthalpies of formation Δ_f_*H*_0_, and constant pressure heat capacities *c*_*p*_0__ of these isomers at temperatures (0–1500) K. These properties are obtained from a correlation developed using statistical mechanics, which have been trained on experimental data.^10^ The experimental data for thermochemical properties of different alkanes available in literature are reported in the NIST chemistry webbook.^11^ Limited amount of experimental data can be found for alkanes longer than C_10_.^12,13^ Thermochemical properties of linear alkanes until C_20_ are listed in refs (14,15). Group contribution methods are commonly used to predict thermochemical properties of long chain alkanes.^16^ These methods are additive in nature, where the structure of a molecule is fragmented into functional groups and the thermochemical properties are estimated by summing up the contributions of each functional group present in the molecule.^16^ The contribution of an individual group remains the same in every molecule it appears.^16^ A wide range of group contribution methods for thermochemical properties of organic molecules exists in literature^17^ which includes methods developed by Benson et al.,^18^ Joback and Reid,^19^ Constantinou and Gani,^20^ Marrero and Gani,^21^ Hukkerikar et al.,^22^ Albahri and Aljasmi,^23^ and Domalski and Hearing.^24^ Benson’s group additivity method^18^ is commonly used to predict Δ_f_*H*_0_ and *S*_0_.^25^ Yaw’s hand book^26^ lists the entropies of formation Δ_f_*S*_0_, Δ_f_*H*_0_, Δ_f_*G*_0_, and *c*_*p*_0__ in gas, liquid, and solid phases for a wide range of alkanes until C_100_. The properties in this hand book are either collected from experimental data available in literature or predicted using Joback and Reid’s method,^19^ especially for long chain alkanes. The Design Institute for Physical Properties (DIPPR) database^27^ also lists the thermochemical properties of a large number of alkanes obtained from quantum chemical calculations, experiments, and group contributions like Benson’s method.^18^ An alternative to predict thermochemical properties is using Machine Learning (ML) models. Yalamanchi et al. predicted Δ_f_*H*_0_ of alkanes, alkenes, and alkynes at 298.15 K using a Support Vector Regression (SVR) model which provided better prediction than Benson’s group additivity when compared with experimental data.^25^ Trinh et al. also used an SVR model to predict Δ_f_*H*_0_ of a wide variety of organic compounds. The training data set was obtained from the DIPPR database.^28^ Aldosari et al. predicted Δ_f_*S*_0_ Δ_f_*H*_0_ and *c*_*p*_0__ of hydrocarbons using SVR, v-SVR, and Random Forest Regression (RFR) algorithms.^29^ Alternatively, one could also consider a High-Dimensional Model Representation (HDMR) for the longer hydrocarbons.^30^ As group contribution methods are more common, we opted for this approach.

**Figure 1:**
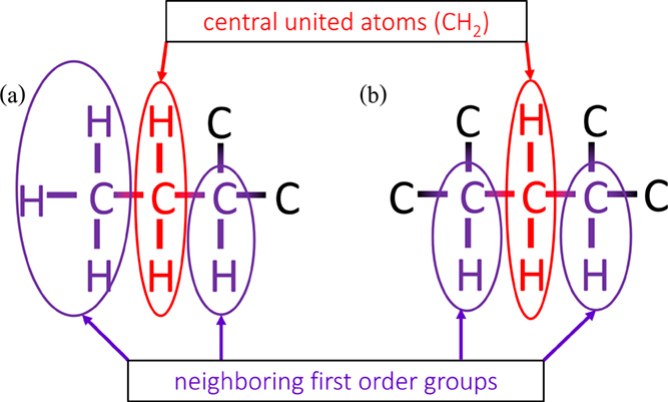
Typical examples of second order groups (a) CH_2_(CH_3_)(CH) and (b) CH_2_(CH)(CH) with CH_2_ as the central united atom. The united atoms inside the brackets are the neighboring first order groups. In first order group contribution methods, only the central united atom is considered which is CH_2_ in cases (a,b). In both cases, first order group contributions will be identical and lead to inaccurate prediction of thermochemical properties. Unlike first order group contribution methods, second order group contribution methods consider the interactions between the central atom (here CH_2_) and the neighboring groups which leads to more accurate prediction of thermochemical properties.

Most of the group contribution methods available in literature predict thermochemical properties for a wide range of hydrocarbons. These methods are not always accurate due to either considering only first order groups (−CH_3_, −CH_2_, −CH, -C) or combining first order group contributions with a very few second order groups.^25^Figure 1 shows typical examples of second order groups (a) CH_2_(CH3)(CH) and (b) CH_2_(CH)(CH) which consider the interaction between the central atom (here CH_2_) and the neighboring united atoms. The united atoms inside the brackets are the first order neighboring groups. Considering only the first order groups leads to less reliable prediction for highly branched isomers. Increasing the number of second order groups in a group contribution method provides a better prediction of the thermochemical properties. Thermochemical property predictions using Scott’s correlation^10^ based on statistical mechanics are in very good agreement with experimental data for chains up to C_10_ isomers. This correlation is very complex and involves many different types of functions and fitting parameters, which makes it hard to apply for long chains (>C_10_) that were not in the training set. ML based predictions have the potential to provide better predictions compared to group contribution methods. However, ML could perform poorly in case of extrapolation^29,31^ which is required for long chain alkanes due to the absence of experimental data. If sufficient number of independent variables are considered and the output is linearly dependent on these variables, Linear Regression (LR) will perform better than the ML models in the extrapolated region.^31^ In this study, an user-friendly LR model is developed, where the occurrences of all possible second order groups are considered as independent variables to accurately predict the thermochemical properties of alkanes longer than C_10_.

Here, we aim to predict the thermochemical properties (*G*_0_ – *H*_0_(0 K)), (*H*_0_ – *H*_0_(0 K)), Δ_f_*G*_0_, and Δ_f_*H*_0_ of alkanes longer than C_10_ isomers using LR with second order groups as molecule descriptors. The training data set includes the ideal gas thermochemical properties of C_1_ to C_10_ isomers at temperatures ranging from (0–1000) K, listed by Scott.^9^ LR is performed at each temperature separately. To account for the effect of temperature, a quadratic polynomial as a function of temperature is fitted to the coefficients of the second order groups for each thermochemical property. The predictions using the second order group contribution method outperform the first order group contribution methods and exceed the chemical accuracy of 1 kcal/mol. This is because the second order groups include the interactions between the neighboring groups of atoms. This study also aims toward using the predicted thermochemical properties Δ_f_*H*_0_ at 0 K and (*G*_0_(*T*) – *H*_0_(0 K)) to compute the reaction equilibrium distribution of hydroisomerization of alkanes longer than C_10_ isomers at that temperature.

To optimize the yield of branched isomers in hydroisomerization, it is important to understand the reaction product distribution at chemical equilibrium.^7,32^ Hydroisomerization reactions involves adsorption of linear alkanes and dehydrogenation of these alkanes in the metal sites of the zeolites forming alkenes.^33^ Protonation of alkenes take place at the acid sites of the zeolites to form alkylcarbenium ions.^34^ These ions are transferred to the metal sites where alkanes are produced via hydrogenation. This indicates that alkenes as intermediates can play an important role at reaction equilibrium. However, due to the absence of alkenes in the final product distribution and lack of experimental data of thermochemical properties of alkenes, the reaction equilibrium of only alkanes is considered in this study. In our previous study, Sharma et al.^7^ studied the shape selectivity effects of zeolites on the reaction equilibrium distribution of hydroisomerization of C_7_ and C_8_ isomers. It was shown that the reaction equilibrium distribution of alkanes is useful for understanding the shape selectivity effects of zeolites on hydroisomerization of alkanes. The reaction equilibrium distribution of this reaction is determined by establishing reaction equilibrium in the gas phase and phase equilibrium between the gas and the adsorbed phase.^7^ For applications such as production of sustainable aviation fuels, long chain alkanes (e.g., C_16_) with high degree of branching^2^ are desirable because of high energy density, low freezing point, and good thermal stability.^35^ The LR model is used to predict Δ_f_*H*_0_ at 0 K and (*G*_0_ – *H*_0_(0 K)) at a specified temperature which are used to compute the gas phase distribution, and classical force field-based simulations are used to quantify the interactions between isomers and the zeolite. To automate the workflow and handling of a large number of isomers, alkanes are represented using SMILES strings.^36,37^ A Python function is developed to generate SMILES strings from the IUPAC names of alkanes which is required as an input in the source code for the LR model (Supporting Information SI2.py). The reaction equilibrium distribution inside constraining pore zeolites differs significantly from the gas phase distribution.^7^ At infinite dilution, the reaction equilibrium distribution is strongly influenced by Henry coefficients of alkanes in constraining pore zeolites such as MTW-type zeolite with pore diameters 5.6 × 6.0 Å^38^ (Figure S1 in the Supporting Information SI5.pdf).^7^ The computation of Henry coefficients using classical force field-based Monte Carlo simulations requires interaction terms as input to account for both bonded and nonbonded interactions of alkanes and also nonbonded interactions between the zeolite atoms and the alkanes. A source code (Supporting Information SI3.py) for automated force field file generation for use with the RASPA2 software^39,40^ is developed to avoid the manual entry of a large number of interaction terms, which is especially useful for long chain alkanes. This code also uses the Python function to generate SMILES strings for alkanes. Using the values for Δ_f_*H*_0_ at 0 K and (*G*_0_ – *H*_0_(0 K)) at 500 K obtained from the LR model and Henry coefficients of alkanes calculated using classical Monte Carlo simulations in the RASPA2 software,^39,40^ the reaction equilibrium distributions of hydroisomerization of C_10_ and C_14_ isomers in MTW-type zeolite at infinite dilution are computed. The reaction equilibrium distribution of C_10_ isomers in MTW-type zeolite computed using the thermochemical properties obtained from our LR model and the training data set are in very good agreement. This suggests that the thermochemical properties predicted using the LR model can be reliably used to compute reaction equilibrium distribution of hydroisomerization of long chain alkanes. In this study, hydroisomerization of C_14_ is shown as an example for long chain alkanes. In future studies, the reaction equilibrium distribution of hydroisomerization of alkanes longer than C_14_ will be analyzed in constraining pore zeolites such as MTW- and MRE-type zeolites.

This article is organized as follows. The important concepts and the theory behind linear regression, and simulation details are provided in Section 2. Our main results are discussed in Section 3. It is observed that LR with second order group contributions outperforms methods based on first order group contributions. The variations in the thermochemical properties due to the differences in branching patterns of isomers are well captured by this method, in sharp contrast to the predictions using group contribution methods available in literature. The coefficients of the occurrences of the second order groups of each thermochemical property are fitted using temperature dependent quadratic polynomials. In Section 4, conclusions on the performance of the LR model in predicting different thermochemical properties of long chain alkanes and the use of these properties for computing reaction equilibrium distribution of hydroisomerization reactions are discussed. The predicted thermochemical properties using this LR model can be reliably used to compute reaction equilibrium distribution of hydroisomerization of alkanes longer than C_10_. This article also contains Supporting Information SI1.xlsx, SI2.py, SI3.py, SI4.xlsx, and SI5.pdf. The training data set for the thermochemical properties of all isomers ranging from C_1_ to C_10_ are listed in SI1.xlsx. In the Supporting Information SI2.py, the source code for the automatic generation of force field files to compute Henry coefficients in the RASPA2 software^39,40^ is included. The source code for the LR model is provided in SI3.py. SI4.xlsx contains the predicted thermochemical properties of all isomers until C_14_ molecules with temperature ranging from (0–1000) K. The coefficients of the occurrences of the second order groups obtained using LR and the corresponding temperature dependent quadratic polynomial fits are also listed in SI4.xlsx. The reaction equilibrium distribution data of hydroisomerization of C_10_ and C_14_ molecules in the gas phase and MTW-type zeolite at 500 K are tabulated in SI4.xlsx. This includes the ideal gas chemical potentials, Henry coefficients, and selectivities of these isomers in both the gas and the adsorbed phase. SI5.pdf contains figures showing the pore structure in MTW-type zeolite, Δ_f_*H*_0_ values from Scott’s tables, our LR model, and the DIPPR database. SI5.pdf also shows the variations of the coefficients of second-order groups with C as the central atom for (*G*_0_ – *H*_0_(0 K)) at different temperatures and the reaction equilibrium distribution imposed on the gas phase for C_10_ isomers.

## 2 Theory

An LR model is used to predict thermochemical properties of alkanes longer than C_10_ with the occurrences of the first or the second order groups as independent variables as shown below. This linear regression is performed using the SciPy library in Python^41^:
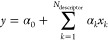
1

In eq 1, *y* is the thermochemical property predicted using LR, *x*_*k*_ is the independent variable which refers to the occurrence of a first or a second order group *k* in a molecule, and *N*_descriptor_ is the total number of such independent variables or descriptors. α_0_ is the intercept of the equation for LR. α_*k*_ refers to the coefficients of the independent variables. In first order group contribution methods, the occurrences of the united atoms CH_4_, CH_3_, CH_2_, CH, and C are considered as independent variables. In second order group contribution methods, the combination of the central united atom and its nearest neighbors is considered as an independent variable. There are 69 second order groups for alkanes which are listed in the Excel worksheet Second_order_grps of the Supporting Information SI1.xlsx. In addition to these groups, CH_4_ is also included in the second order group contribution to predict the thermochemical properties of methane.

The training data set contains isomers in the range C_1_–C_10_. This data set includes the occurrences of only 46 second order groups. The remaining second order groups are present in alkanes of chain length longer than C_10_. The list of the second order groups which are not present in the training data set along with the smallest isomers which contain these groups are shown in the worksheet Second_order_grps of the Supporting Information SI1.xlsx. To account for the contribution of these groups in the longer chains, these groups are approximated by a similar second order group present in the training data set. For example, the second order group C(C)(CH_2_)(CH_2_)(CH_2_) is approximated by C(C)(CH_2_)(CH_2_)(CH_3_). The list of all such approximations are also included in the Excel worksheet Second_order_grps of the Supporting Information SI1.xlsx. These second order groups correspond to highly branched isomers which are unlikely to form inside constraining pore zeolites like MTW-type zeolite during hydroisomerization of alkanes. This is due to constraining pores present in MTW-type zeolite and steric hindrance caused by the proximity of the branches present in these isomers.^7^ Therefore, approximate predictions of the thermochemical properties of such alkanes are sufficient to compute the reaction equilibrium distribution of all hydroisomerization reactions inside zeolites.

Linear regressions for the thermochemical properties are performed at a specific temperature and the coefficients of the second order groups (α_*k*_) obtained from this model are specific to that temperature. To include the effect of temperature, these coefficients are refitted to a temperature dependent quadratic polynomial.
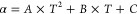
2

In eq 2, *A*, *B*, and *C* are constants and *T* is the temperature in K. The values of the coefficients for the thermochemical properties Δ_f_*H*_0_, Δ_f_*G*_0_, (*G*_0_ – *H*_0_(0 K)), and (*H*_0_ – *H*_0_(0 K)) are listed in the Excel worksheets DHf0_coeff, DGf0_coeff, G0-H0(0K)_coeff, and H0–H0(0K)_coeff of the Supporting Information SI2.xlsx, respectively. The corresponding temperature dependent polynomials are listed in the Excel worksheets DHf0_coeff_poly, DGf0_coeff_poly, G0-H0(0K)_coeff_poly, and H0–H0(0K)_coeff_poly of SI4.xlsx. The source code to predict thermochemical properties of alkanes using LR is provided in the Supporting Information SI2.py. To handle a large number of isomers during linear regression, alkanes are represented as SMILES strings.^36,37^ This code includes a function to generate SMILES strings for alkanes with maximum of 5-carbon alkyl branches. The thermochemical properties, Δ_f_*H*_0_ at 0 K and (*G*_0_ – *H*_0_(*T*_ref_)) at a specified temperature are used to compute ideal gas chemical potentials^42−44^ which are further used to compute the reaction equilibrium distribution of hydroisomerization of alkanes. The ideal gas chemical potential of component *i* equals^42,43^
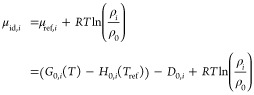
3

In eq 3, μ_ref,*i*_ is the reference chemical potential of component *i*, ρ_*i*_ is the number density of component *i*, and ρ_0_ is the reference density which is chosen to be 1 molecule/Å^3^. *T*_ref_ is the reference temperature which is 0 K in this study. *D*_0,*i*_ is the atomization energy^44^ which is
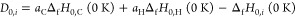
4

In eq 4, *a*_C_ is the number of C atoms and *a*_H_ is the number of H atoms present in the alkane isomer *i*. Δ_f_*H*_0,C_, Δ_f_*H*_0,H_, and Δ_f_*H*_0,*i*_ are the enthalpies of formation of the C atom, the H atom, and alkane isomer *i*, respectively, at 0 K. The value of Δ_f_*H*_0,C_ is 711.185 kJ/mol and Δ_f_*H*_0,H_ is 216.035 kJ/mol at 0 K which are obtained from the JANAF tables.^45^ Δ_f_*H*_0,*i*_ values of alkanes are provided in the Supporting Information SI1.xlsx.

The reaction equilibrium distribution of hydroisomerization reactions in zeolites can be computed by imposing reaction equilibrium in the gas phase and phase equilibrium between the gas and the adsorbed phase.^7,32,46^ At reaction equilibrium, the chemical potentials of the reactants and the reaction products are equal in the gas phase.^7^ Equating the ideal gas chemical potentials of the reactants and the reaction products leads to the gas phase reaction equilibrium distribution at infinite dilution. The equilibrium loadings in the adsorbed phase are computed using Henry’s law at infinite dilution and mixture adsorption isotherm models such as Ideal Adsorbed Solution Theory (IAST)^47,48^ at finite loadings. At high temperatures (≥500 K), the effect of pressure on the reaction equilibrium distribution of hydroisomerization of alkanes is negligible.^7^ High temperature leads to negligible variations in the gas phase distribution of alkane isomers with pressure and a decrease in the amount of molecules adsorbing in the zeolites.^7^ Therefore, only infinite dilution is considered in this study. For details on computing reaction equilibrium distribution of hydroisomerization, the reader is referred to our previous study.^7^

The required Henry coefficients are computed using the Widom test particle insertion method^49^ combined with the Configurational-Bias Monte Carlo (CBMC) method^50−53^ in the RASPA2 software.^39,40^ Alkanes are modeled using a united-atom model^54^ and the Coulomb interactions are neglected because alkanes are nonpolar.^39^ The intramolecular nonbonded interactions of alkanes and the intermolecular nonbonded interactions between the alkanes and the zeolite atoms are modeled using the Lennard-Jones interactions.^55^ The Lennard-Jones parameters for alkanes are obtained from Dubbeldam et al.^56^ The Lennard-Jones parameters for the zeolite atoms are taken from the TraPPE-zeo force field.^57^ The intramolecular bonded interactions which include bond-stretching, bond-bending, and torsion interactions are obtained from refs (58,59). Both bonded and nonbonded interaction parameters are listed in the Excel worksheet force field_param of the Supporting Information SI1.xlsx. Files containing force field parameters and the list of bonded and nonbonded interactions are required as input in the RASPA2 software.^39,40^ The number of intramolecular interactions increases tremendously with increasing chain lengths of alkanes (e.g., n-C_14_ contains 91 intramolecular interactions). Therefore, a Python code for automatic generation of force field files for alkanes is provided in the Supporting Information SI3.py. This code requires alkanes to be represented as SMILES strings.^36,37^ Therefore, this code also includes the Python function to generate SMILES strings for alkanes. All Lennard-Jones interactions are truncated and shifted at 12 Å without applying tail corrections. The number of unit cells in the simulation box of MTW-type zeolite are 2 × 15 × 2 for C_10_ isomers and 2 × 25 × 2 for C_14_ isomers. The zeolite is considered to be a rigid structure as the effect of zeolite flexibility is negligible on adsorption processes, especially at infinite dilution.^60^

The selectivity of a component in the gas phase or the adsorbed phase is computed as^61^
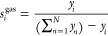
5

The selectivity of a component (eq 5) in both gas phase and adsorbed phase is defined as the ratio of the mole fraction *y*_*i*_ of the component to the sum of the mole fractions of all other components present in the same phase. To compare the selectivity of a component relative to another, the term relative selectivity (*s*_rel,*i*_) is defined as the ratio of the absolute selectivity of that component to a reference component as shown in eq 6 for both gas and adsorbed phase.
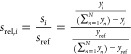
6

In this study, n-C_10_ and n-C_14_ molecules are chosen as reference components for computing relative selectivities.

## 3 Results and Discussion

**Figure 2:**
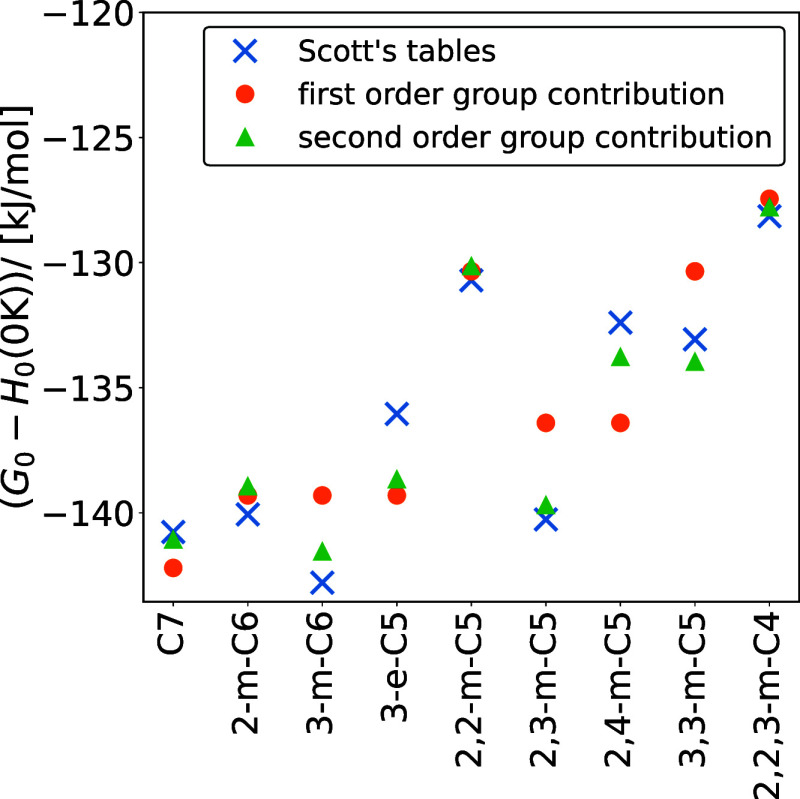
Comparison between the thermochemical property (*G*_0_ – *H*_0_(0 K)) predicted using the LR model with first and second order group contribution methods for C_7_ isomers at 298.15 K. The predictions using the second order group contributions are in very good agreement with the data from the Scott’s tables.^9^

Figure 2 shows the comparison between (*G*_0_ – *H*_0_(0 K)) of C_7_ isomers at 400 K predicted by the LR model using first and second order group contribution methods. The predicted values are also compared with the data obtained from the tables by Scott.^9^ The thermochemical properties obtained using the second order groups as descriptors are in very good agreement with the training data set (Figure 2) These predictions are much better than those obtained using the first order group contributions. This is because the influence of the neighboring groups of atoms are neglected in the first order group contribution which is clearly shown in Figure 2.

The Mean Absolute Errors (MAEs) for the prediction of the thermochemical properties of alkanes using the first and the second order group contributions are listed in Table 1. The use of the second order groups as descriptors leads to smaller MAEs compared to the first order group contributions. Therefore, only second order group contributions are considered in this study. For comparison, the values of Δ_f_*H*_0_ at 400 K for a few selected C_10_ isomers obtained from the training data set^9^ and computed using the LR model are listed in Table 2. The values computed using the LR model and those obtained from the training data set are in excellent agreement.

**Table 1:** Mean Absolute Errors (MAEs) of the Thermochemical Properties Δ_f_*G*_0_, Δ_f_*H*_0_, (*G*_0_ – *H*_0_(0 K)), (*H*_0_ – *H*_0_(0 K)) Predicted Using Linear Regression (LR) with the First and the Second Order Group Contributions

thermochemical property	MAE (first order)/(kJ/mol)	MAE (second order)/(kJ/mol)
Δ_f_*G*_0_	7.529	1.029
Δ_f_*H*_0_	5.834	0.152
(*G*_0_ – *H*_0_(0 K))	3.087	1.012
(*H*_0_ – *H*_0_(0 K))	1.041	0.181

**Table 2:** Comparison between the Values of Enthalpy of Formation Δ_f_*H*_0_ for C_10_ Isomers at 400 K Obtained from the Training Dataset^9^ and the Linear Regression Model

isomer	Δ_f_*H*_0_/(kJ/mol)	
	training data	linear regression data
n-C_10_	–265.47	–265.47
4-m-C_9_	–270.04	–270.07
4-e-C_8_	–267.48	–267.49
2,2-m-C_8_	–282.55	–282.56
3-e-4-m-C_7_	–266.60	–266.53
2,2,5-m-C_7_	–287.19	–287.09
3,3-e-C_6_	–266.10	–266.08
4-e-3,3-m-C_6_	–261.75	–261.76
2,2,3,5-m-C_6_	–284.68	–284.57
3-e-2,2,4-m-C_5_	–261.37	–261.37
2,2,3,4,4-m-C_5_	–261.54	–261.54

**Figure 3:**
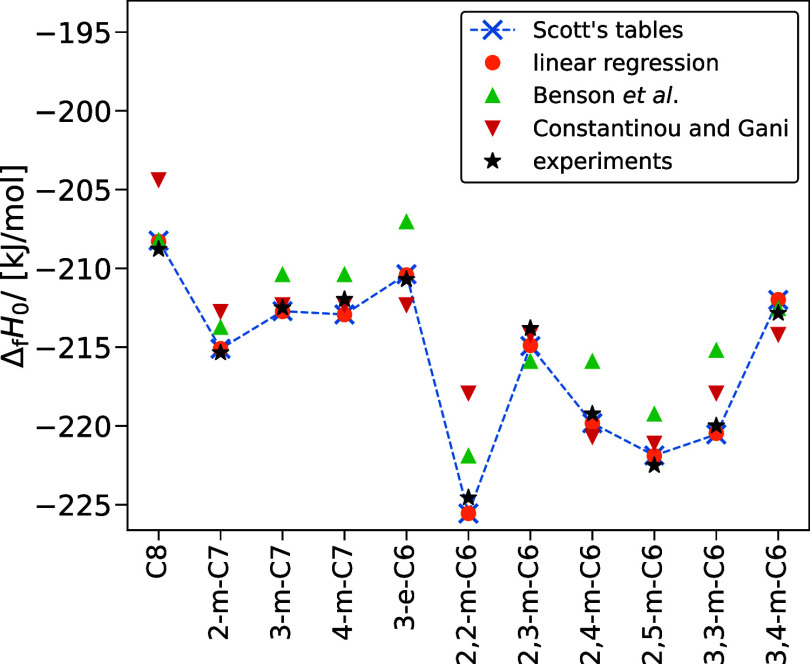
Prediction of Δ_f_*H*_0_ for C_8_ isomers at 298.15 K using the LR model, Benson et al.’s group additivity method,^18^ Constantinou and Gani’s group contribution,^20^ the Scott’s tables,^9^ and the experimental data listed by Scott.^10^ The predictions using the LR model are in excellent agreement with the Scott’s tables and the data from the experiments^10^ For the Δ_f_*H*_0_ values of C_8_ isomers at 298.15 K, using the experimental data as the reference, the Mean Absolute Errors (MAEs) are the smallest for our LR model (0.62 kJ/mol) and the Scott’s Tables (0.64 kJ/mol). This is followed by Constantinou and Gani’s group contribution method (2.04 kJ/mol), and Benson et al.’s group additivity method (2.37 kJ/mol) respectively. The dashed blue line through the data points obtained from the Scott’s tables is a guide to the eye.

Several group contribution methods are available in literature such as Benson’s,^18^ Constantinou and Gani’s,^20^ Joback’s,^19^ and Domalski and Hearing’s^24^ methods which either consider first order group contributions or a combination of first order groups and a few second order groups. Figure 3 shows the variations in Δ_f_*H*_0_ of a few C_9_ isomers at 298.15 K predicted using the LR model, Benson’s,^18^ and Constantinou and Gani’s methods.^20^ The properties predicted using Benson’s and Constantinou and Gani’s methods are computed using the SPLIT software by AmsterChem.^62^Figure 3 also includes data obtained from the Scott’s tables,^9^ and Yaws’ handbook.^26^ Yaws’ handbook^26^ uses Joback’s group contribution method^19^ for long chain isomers. Benson’s^18^ and Constantinou and Gani’s^20^ group contribution methods are not always able to distinguish between isomers based on the positions and the types of branches these isomers possess. For example, the Constantinou and Gani’s method^20^ provided nearly identical values of Δ_f_*H*_0_ for 3-m-C_8_, 4-m-C_8_, 3-e-C_7_, and 4-e-C_7_ whereas experimental data from Scott’s tables clearly shows variations due to the presence of the methyl and the ethyl groups as branches in these isomers. Similarly, Benson’s method^18^ does not distinguish between 2,3-m-C_7_, 2,4-m-C_7_, and 2,5-m-C_7_. Such variations are well captured by our LR model and are necessary to determine the selectivities of reaction products in hydroisomerization of alkanes.^7^ Δ_f_*H*_0_ values of a few C_9_ and C_10_ isomers at 298.15 K obtained from the Scott’s tables and predicted by LR are also compared with those listed in the DIPPR database^27^ as shown in Figure S2 in the Supporting Information SI5.pdf. Δ_f_*H*_0_ of C_10_ isomers listed in the DIPPR database^27^ are computed using the Domalski and Hearing’s method.^24^ The values predicted using our LR model are in excellent agreement with the experimental data from the Scott’s tables. The values obtained from the DIPPR database^27^ are also in good agreement with the Scott’s tables^9^ with small deviations for 4-m-C_8_, 2-m-C_9_, and 3-m-C_9_.

LR is performed at a specific temperature for each thermochemical property. To account for the effect of temperature on the thermochemical properties and compute these properties at any temperature, the coefficients which correspond to the occurrence of the second order groups are fitted to a temperature dependent quadratic polynomial (eq 2). Figure S3 in the Supporting Information SI5.pdf shows the variations in the magnitudes of the coefficients of the occurrences of the second order groups for (*G*_0_ – *H*_0_(0 K)) with C as the center atom present in the training data set. The fitted coefficients using the quadratic polynomial are in excellent agreement with those predicted using the LR model. The values of the coefficients are different at a specific temperature (Figure S3 in the Supporting Information SI5.pdf). In case of first order group contributions, the variations will be identical as each group has the same central united atom C. Therefore, combining these coefficients into a single coefficient to reduce the number of independent variables or simply using first order group contributions will lead to erroneous predictions of the thermochemical properties. This clearly indicates the need for a second order group contribution method.

**Figure 4:**
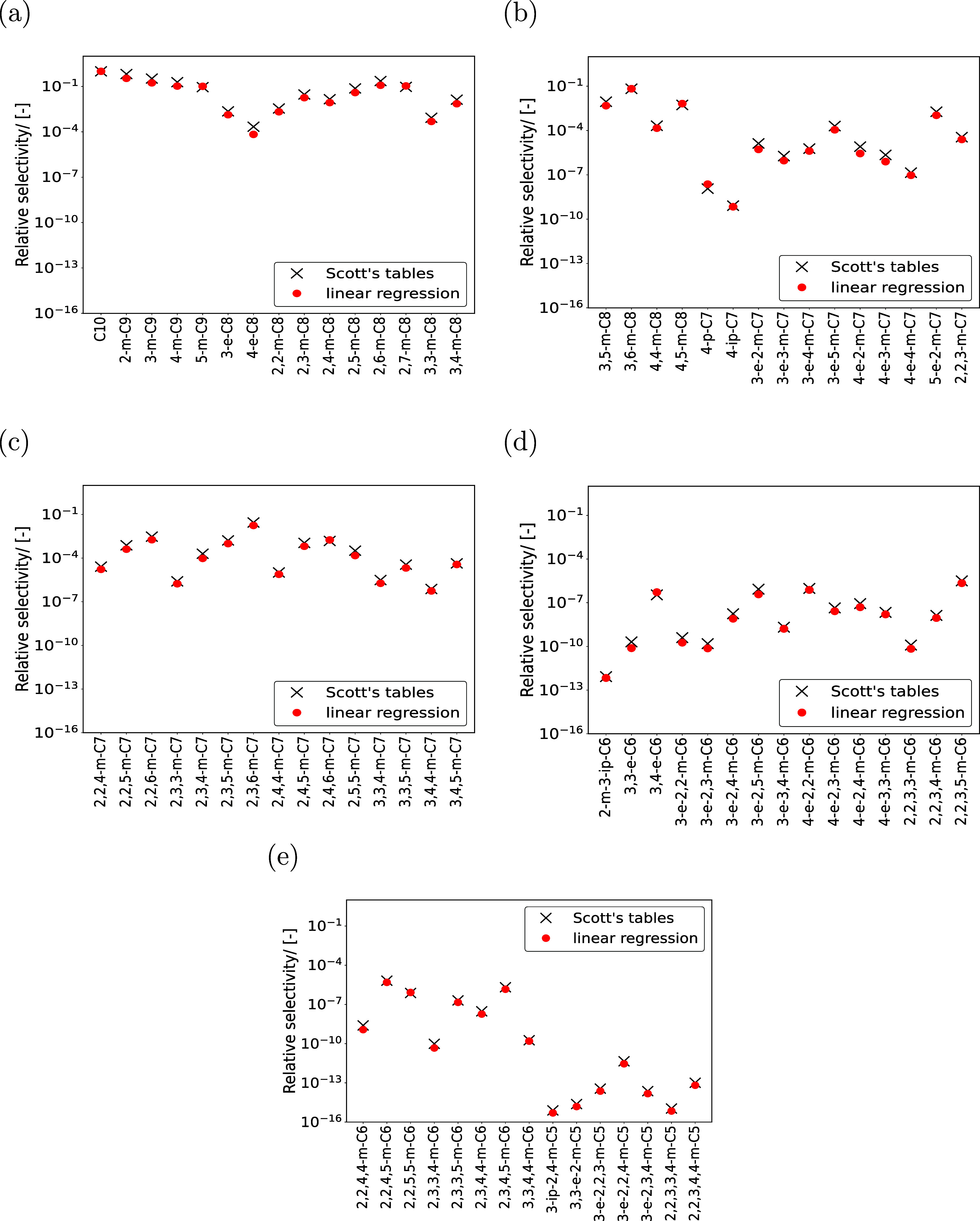
Selectivities of C_10_ isomers relative to n-C_10_ at reaction equilibrium in MTW-type zeolite at infinite dilution and 500 K. The gas phase reaction equilibrium distribution required in computing the adsorbed phase reaction equilibrium distribution are obtained using the Scott’s tables (black crosses) and the LR model (red filled circles). The raw data is listed in the Excel worksheet xi_iC10_500 K of the Supporting Information SI4.xlsx.

The predicted thermochemical properties (*G*_0_ – *H*_0_(0 K)) at a specified temperature and Δ_f_*H*_0_ at 0 K for alkanes longer than C_10_ are used to compute the ideal gas chemical potentials which are further used in calculating the reaction equilibrium distribution of hydroisomerization of long chain alkanes. Figure S4 in the Supporting Information SI5.pdf shows the reaction product distribution of C_10_ isomers in the gas phase at infinite dilution and 500 K. The reaction product distribution obtained using LR is in very good agreement with the training data set (Figure S4 in the Supporting Information SI5.pdf). In both cases, the variations in the selectivities of C_10_ isomers relative to n-C_10_ are similar. The gas phase distribution and Henry coefficients are used to compute the reaction equilibrium distribution in MTW-type zeolite at infinite dilution and 500 K (Figure 4). For monobranched isomers, 4-p-C_7_ and 4-ip-C_7_ have the smallest preferences in MTW-type zeolite as shown in Figure 4b. This indicates that isomers with branches longer than propyl or isopropyl groups such as butyl and pentyl groups will have even lower selectivities in MTW-type zeolite. Such isomers can be eliminated form the calculation of the reaction equilibrium distribution of hydroisomerization of long chain alkanes. The ethyl-trimethyl isomers (3-e-2,2,3-m-C_5_, 3-e-2,2,4-m-C_5_, and 3-e-2,3,4-m-C_5_) and pentamethyl isomers (2,2,3,3,4-m-C_5_ and 2,2,3,4,4-m-C_5_) have the least preference compared to all other isomers. This indicates that isomers with multiple branches present in the vicinity of each other are not preferably formed inside constraining pore zeolites such as MTW-type zeolite. Reaction product distributions obtained using both LR and Scott’s tables provide very similar variations in selectivities. This suggests that the thermochemical properties obtained using LR are reliable and can be used in computing the reaction equilibrium distribution for hydroisomerization of long chain alkanes.

**Figure 5:**
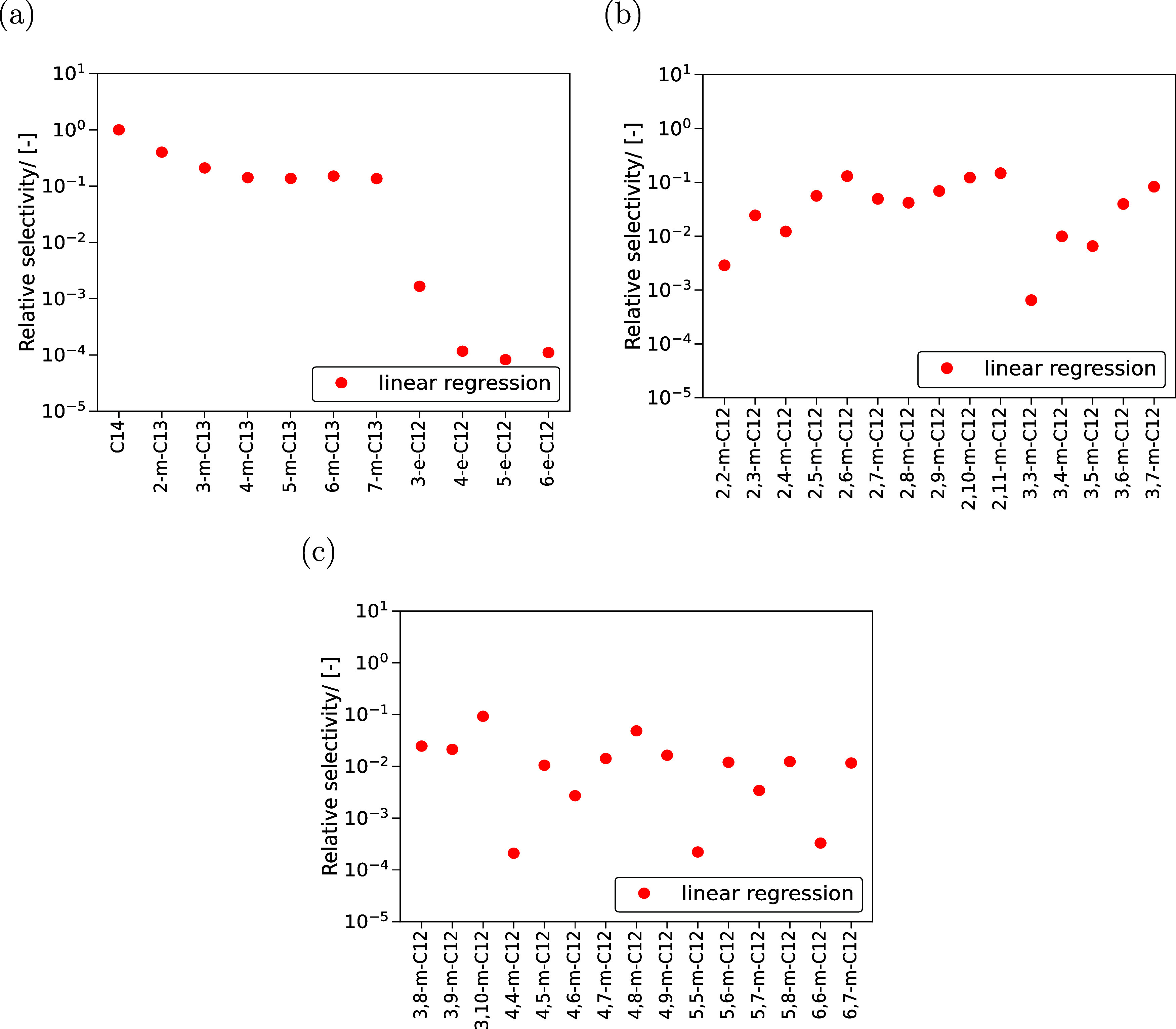
Selectivities of different C_14_ isomers relative to n-C_14_ at reaction equilibrium in MTW-type zeolite at infinite dilution and 500 K. The LR model is used to predict (*G*_0_ – *H*_0_ (0 K)) at 500 K and Δ_f_*H*_0_ at 0 K which are used to compute the ideal gas chemical potentials of C_14_ isomers. These chemical potentials are used to compute the gas phase reaction equilibrium distribution of hydroisomerization of C_14_. The raw data is listed in the Excel worksheet xi_iC14_500 K of the Supporting Information SI4.xlsx.

Figure 5 shows the reaction product distribution of C_14_ isomers in MTW-type zeolite at infinite dilution and 500 K. Monomethyl branched isomers have higher preference compared to the monoethyl and dimethyl isomers. This is because of the smaller pore diameters present in MTW-type zeolites (5.6 × 6.0) Å.^38^ The order of magnitude of relative selectivities varies for dibranched isomers which depends on the proximity of the methyl branches. The geminal alkanes (2,2-m-C_12_, 3,3-m-C_12_, 4,4-m-C_12_, 5,5-m-C_12_, and 6,6-m-C_12_) have the least selectivity due to steric hindrance posed by the presence of two methyl groups at the same position in the alkane chain. Isomers with methyl groups far apart (2,10-m-C_12_ and 2,11-m-C_12_) have the highest selectivities compared to other dimethyl isomers. In future studies, such criteria will be used to identify relevant isomers for computing reaction equilibrium distribution of alkanes longer than C_14_. Such filtering of isomers is necessary because the number of isomers increases enormously for alkanes longer than C_14_ and it is difficult to consider each isomer experimentally.

## 4 Conclusions

A linear regression model with second order group contributions is developed to predict the thermochemical properties Δ_f_*H*_0_, Δ_f_*G*_0_, (*G*_0_ – *H*_0_(0 K)), and (*H*_0_ – *H*_0_(0 K)) of alkanes. The predicted properties are in excellent agreement with the Scott’s tables^9^ and exceed the chemical accuracy of 1 kcal/mol. The maximum mean absolute error is observed for Δ_f_*G*_0_ which is 1.03 kJ/mol. The second order group contribution method outperforms first order group contribution methods for predicting these properties. Our LR model performs better than many existing group contribution methods in literature such as the Benson’s,^18^ Constantinou and Gani’s,^20^ and Joback’s methods.^19^ The influence of temperature is considered by fitting the coefficients of the occurrences of the second order groups with a temperature dependent quadratic polynomial. The reaction equilibrium distributions computed using the LR model and the Scott’s tables^9^ in combination with automatic computation of Henry coefficients from RASPA2^39,40^ are in very good agreement for the hydroisomerization of C_10_ isomers in MTW-type zeolite. This indicates that the LR model can be used to compute reaction equilibrium distribution of long chain alkanes in zeolites. The isomers with propyl and the isopropyl groups as branches have low selectivity in MTW-type zeolites. This indicates that isomers with branches longer than propyl group such as butyl or pentyl groups will have very low selectivity and can be neglected from reaction product distribution. The reaction equilibrium distribution for the hydroisomerization of C_14_ isomers in MTW-type zeolite is also computed using the thermochemical properties predicted by the LR model. Isomers with branches far apart (e.g., 2,10-m-C_12_ and 2,11-m-C_12_) have larger selectivities compared to isomers with branches present close to each other (2,2-m-C_12_ and 3,3-m-C_12_). Such criteria are necessary to exclude isomers with very low selectivities from the analysis of reaction equilibrium distributions of alkanes longer than C_14_. In future studies, hydroisomerization of alkanes longer than C_14_ will be considered for which the thermochemical properties will be computed using our LR model. As this involves a large number of isomers, automatic generation of force field files and other input files for RASPA2 will be essential (SI3.py).

## Supporting Information Available

Contains Supporting Information (SI1.xlsx, SI2.py, SI3.py, SI4.xlsx, and SI5.pdf); training data sets of the thermochemical properties of all isomers ranging from C_1_ to C_10_ listed in SI1.xlsx; list of the second-order groups present in the training data set listed in the Supporting Information (SI1.xlsx); force field parameters to compute Henry coefficients of alkanes in zeolite listed in SI1.xlsx; source code for the LR model listed in SI2.py; code provides thermochemical properties of alkanes directly from the SMILES string; source code for automated force field file generation, using the SMILES string as input, of branched alkanes for use with the RASPA2 software provided in SI3.py; predicted thermochemical properties of all alkane isomers until C_14_ for temperatures ranging from (0–1000) K listed in SI4.xlsx; linear regression coefficients of the second-order groups and the corresponding temperature-dependent quadratic polynomial fits also listed in SI4.xlsx; and reaction equilibrium distribution data of hydroisomerization of C_10_ and C_14_ in the gas phase and MTW-type zeolite at 500 K tabulated in SI4.xlsx (ZIP)
SI5.pdf contains figures showing the pore structure in MTW-type zeolite, Δ_f_*H*_0_ values obtained from the Scott’s tables, our LR model, and DIPPR database, and the coefficients of second-order groups with C as the central atom at different temperatures for (*G*_0_ – *H*_0_(0 K)); reaction equilibrium distribution imposed on the gas phase for hydroisomerization of C_10_ isomers also shown in SI5.pdf (PDF)
