# Supplementary material for: Prediction of Thermochemical Properties of Long-Chain Alkanes Using Linear Regression: Application to Hydroisomerization
Source: J Phys Chem B. 2024 Sep 23;128(39):9619–29. doi: 10.1021/acs.jpcb.4c05355 (PMC11457146; doi:10.1021/acs.jpcb.4c05355)
Supplement: Supplementary file 2 — jp4c05355_si_002.pdf [file jp4c05355_si_002.pdf]

Supporting Information for: Prediction of Thermochemical Properties of Long-Chain Alkanes using Linear Regression: Application to Hydroisomerization

Shrinjay Sharma,[†] Josh J. Sleijfer,[‡,¶] Jeroen Op de Beek,[‡] Stach van der Zeeuw,[¶] Daniil Zorzos,[§] Silvia Lasala,[∥] Marcello S. Rigutto,[⊥] Erik Zuidema,[⊥] Umang Agarwal,[#] Richard Baur,[⊥] Sofia Calero,[@] David Dubbeldam,[△] and Thijs J.H. Vlugt[*,†]

†*Engineering Thermodynamics, Process & Energy Department, Faculty of Mechanical Engineering, Delft University of Technology, Delft, The Netherlands*

‡*Delft Institute of Applied Mathematics, Faculty of Electrical Engineering, Mathematics and Computer Science, Delft University of Technology, Delft, The Netherlands*

¶*Faculty of Applied Sciences, Delft University of Technology, Delft, The Netherlands*

§*Faculty of Aerospace Engineering, Delft University of Technology, Delft, The Netherlands*

∥*Université de Lorraine, CNRS, Nancy, France*

⊥*Shell Global Solutions International B.V., Amsterdam, The Netherlands*

#*Shell Chemical LP, Monaca, The United States*

@*Department of Applied Physics, Eindhoven University of Technology, Eindhoven, The Netherlands*

△*Van 't Hoff Institute of Molecular Sciences, University of Amsterdam, Amsterdam, The Netherlands*

E-mail: t.j.h.vlugt@tudelft.nl

The following items are presented in this Supporting Information:

- Schematic representation of the pore structure of MTW-type zeolite.
- Comparison between $\Delta_f H_0$ values at 298.15 K obtained from Linear Regression (LR), Scott's tables,[1] and the DIPPR database.[2]
- Variations of the coefficients of second order groups with C as the central atom at different temperatures.
- Reaction equilibrium distribution of C_{10} isomers relative to n-C_{10} in the gas phase at infinite dilution and 500 K.

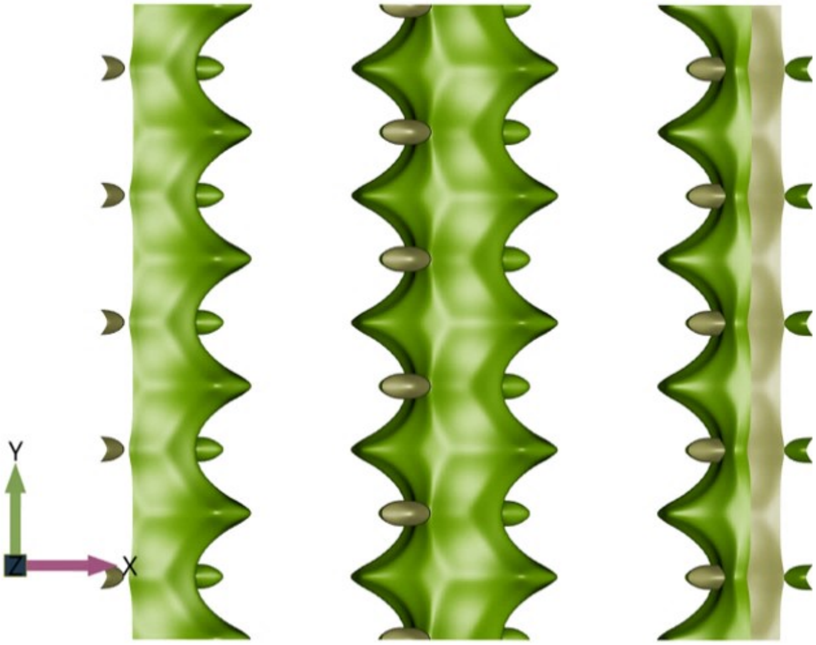

Figure S1: Schematic representation of the pore structure of MTW-type zeolite. This zeolite contains one dimensional channel-like pores in vertical direction (y-axis) with pore diameters 5.6×6.0 Å[3] The iRASPA software[4] is used to generate these images.

Fig. S1 shows the one dimensional pore structure of MTW-type zeolite obtained using the iRASPA software.[4] The reaction equilibrium distributions of hydroisomerization of C_{10} and C_{14} isomers are studied inside the pores of MTW-type zeolite.

Fig. S2 shows the comparison between $\Delta_f H_0$ of C_9 and C_{10} isomers at 298.15 K obtained from the Scott's tables,[1] predictions by our LR model, and those listed in the DIPPR database.[2] $\Delta_f H_0$ of C_{10} isomers (Fig. S2) listed in the DIPPR database[2] are computed using the Domalski and Hearing's method.[5] The values predicted using our LR model are in excellent agreement with the experimental data from the Scott's tables. The values obtained from the DIPPR database[2] are also in good agreement with the Scott's tables[1] with small deviations for 4-m-C_8, 2-m-C_9, and 3-m-C_9.

Fig. S3 shows the variations in the coefficients of the second order groups for $(G_0 - H_0(0\text{ K}))$ with C as the centre atom present in the training dataset at different temperatures. The fitted coefficients using the quadratic polynomial are in excellent agreement with those pre-

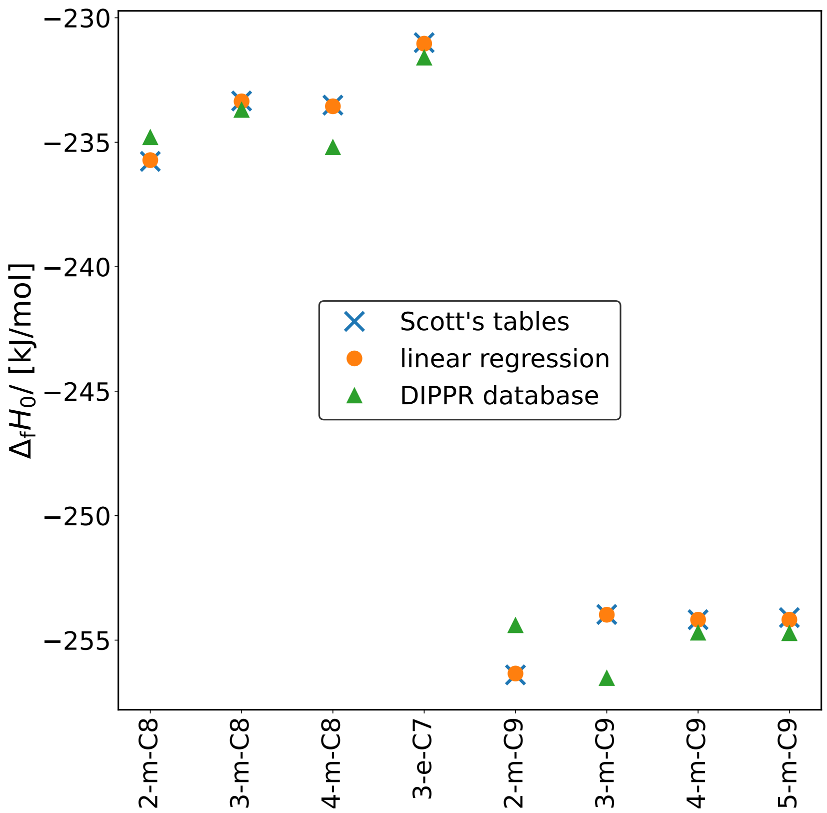

Figure S2: Comparison between the predictions of $\Delta_f H_0$ at 298.15 K for C_9 and C_{10} isomers using the LR model and the data obtained from the DIPPR database.[2] The predictions using the LR model are in excellent agreement with the data obtained from the Scott's tables[1]

dicted using the LR model. In case of first order group contributions, all entries will be identical as each group has the same central united atom C. Therefore, combining these coefficients into a single coefficient to reduce the number of independent variables or simply using first order group contributions will lead to erroneous predictions of the thermochemical properties. This clearly indicates the need for a second order group contribution method.

Fig. S4 shows the reaction equilibrium distribution of C_{10} isomers relative to n-C_{10} imposed in the gas phase at infinite dilution and 500 K. The reaction equilibrium distribution obtained using the Scott's tables[1] and our LR model are in very good agreement. The gas phase distribution combined with phase equilibrium between the gas and the adsorbed

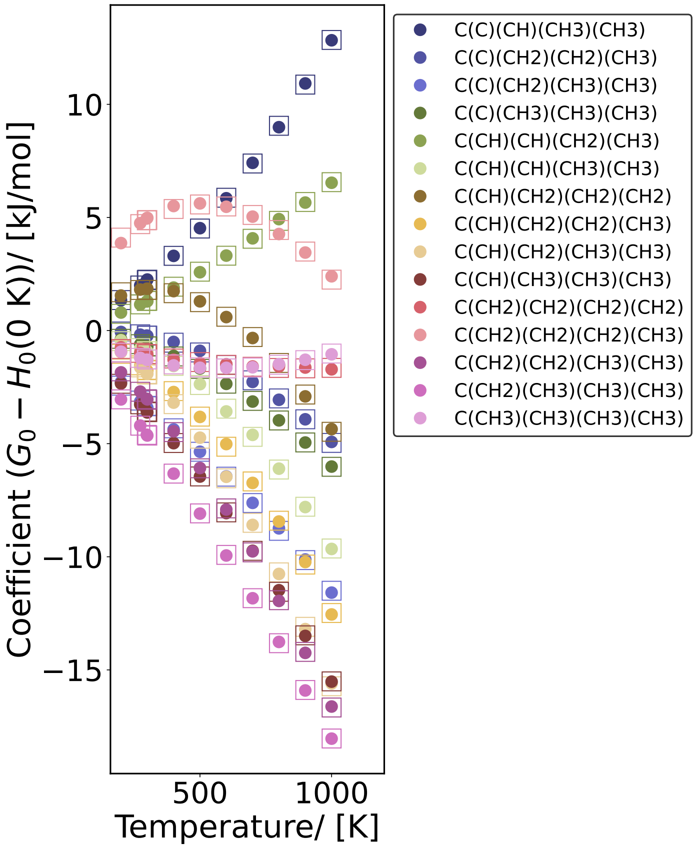

Figure S3: Variations of the coefficients of second order groups with C as the central atom at different temperatures. This plot includes all second order groups with C as the central atom present in the training dataset[1] for the thermochemical property ($G_0 - H_0(0\ \mathrm{K})$). The colored circles represent the coefficients of the second order groups obtained using linear regression and the square symbols are for those obtained using the temperature dependent quadratic polynomial fit. The quadratic polynomial is fitted to the coefficients obtained using the LR model.

phases provide the reaction equilibrium distribution inside the pores of MTW-type zeolite for the hydroisomerization reaction (Figs. 4 and 5 in the main text).

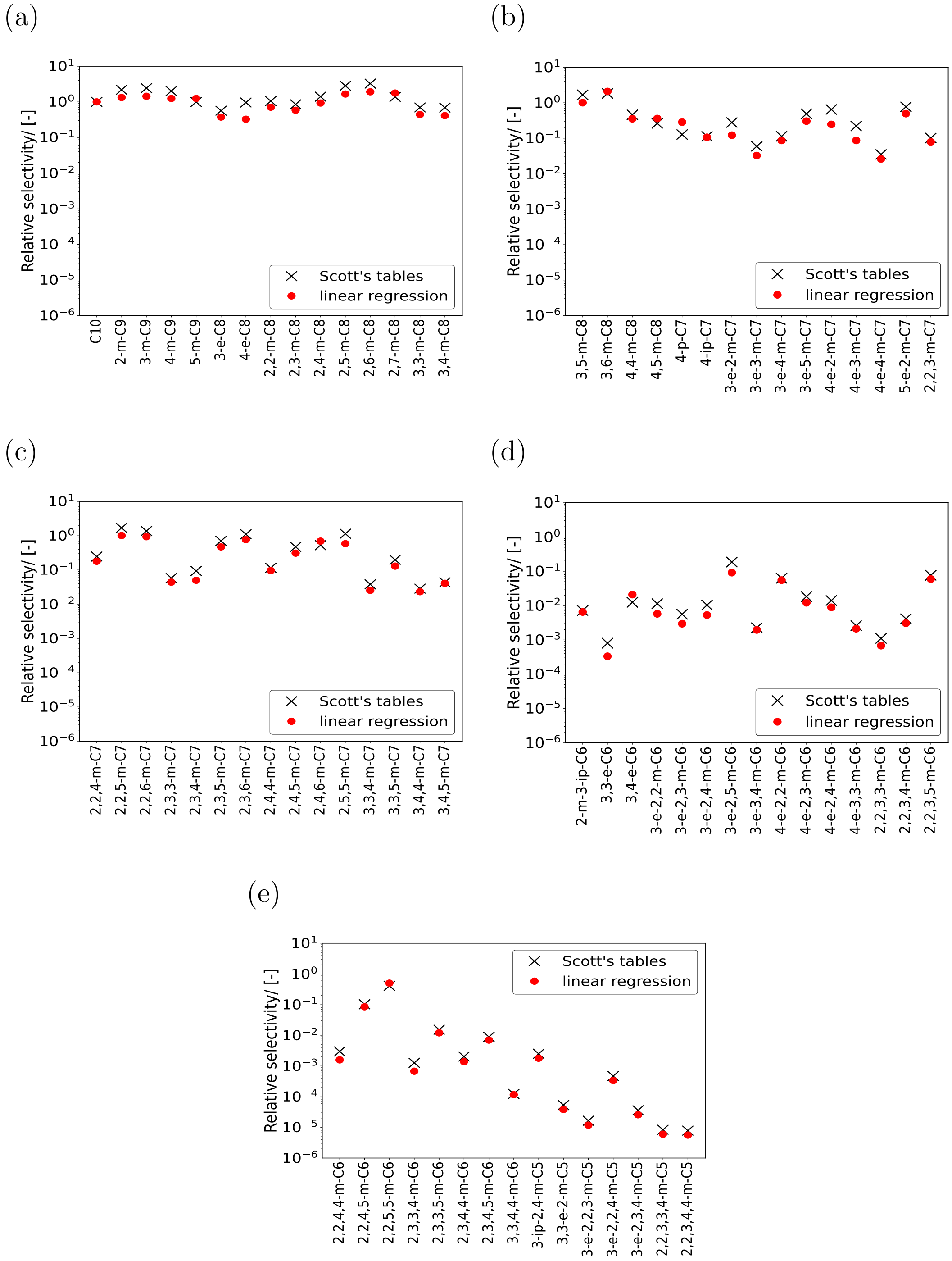

Figure S4: Gas phase distribution of C_{10} isomers relative to n-C_{10} at reaction equilibrium in the gas phase at infinite dilution and 500 K. Both Scott's tables (black crosses) and the LR model (red filled circles) are used to predict $(G_0 - H_0(0\text{ K}))$ at 500 K and $\Delta_f H_0$ at 0 K which are used to compute the ideal gas chemical potentials of C_{10} isomers. These chemical potentials are used to compute the gas phase reaction equilibrium distribution of hydroisomerization of C_{10}.[6] The reaction equilibrium distribution obtained using the Scott's tables and the LR model are in very good agreement. The raw data is listed in the Excel worksheet xi_iC10_500K of the Supporting Information SI4.xlsx.

References

(1) Scott, D. W. *Chemical thermodynamic properties of hydrocarbons and related substances: properties of the alkane hydrocarbons, C_1 through C_{10}, in the ideal gas state from 0 to 1500 K*, 1st ed.; US Department of the Interior, Bureau of Mines: Washington DC, 1974.

(2) Bloxham, J. C.; Redd, M. E.; Giles, N. F.; Knotts IV, T. A.; Wilding, W. V. Proper use of the DIPPR 801 database for creation of models, methods, and processes. *Journal of Chemical & Engineering Data* **2020**, *66*, 3–10.

(3) Baerlocher, C.; McCusker, L. B.; Olson, D. H. *Atlas of zeolite framework types*, 6th ed.; Elsevier: Amsterdam, 2007.

(4) Dubbeldam, D.; Calero, S.; Vlugt, T. J. H. iRASPA: GPU-accelerated visualization software for materials scientists. *Molecular Simulation* **2018**, *44*, 653–676.

(5) Domalski, E. S.; Hearing, E. D. Estimation of the Thermodynamic Properties of Hydrocarbons at 298.15 K. *Journal of Physical and Chemical Reference Data* **1988**, *17*, 1637–1678.

(6) Sharma, S.; Rigutto, M. S.; Zuidema, E.; Agarwal, U.; Baur, R.; Dubbeldam, D.; Vlugt, T. J. H. Understanding shape selectivity effects of hydroisomerization using a reaction equilibrium model. *Journal of Chemical Physics* **2024**, *160*, 214708.
